# The Significant Role of Tumor Volume on the Surgical Approach Choice, Surgical Complexity, and Postoperative Complications in Renal Cell Carcinoma With Venous Tumor Thrombus From a Large Chinese Center Experience

**DOI:** 10.3389/fonc.2022.869891

**Published:** 2022-06-07

**Authors:** Qais Baheen, Zhuo Liu, Yichang Hao, Rejean R. R. Sawh, Yuxuan Li, Xun Zhao, Peng Hong, Zonglong Wu, Lulin Ma

**Affiliations:** Department of Urology, Peking University Third Hospital, Beijing, China

**Keywords:** tumor volume (TV), renal cell carcinoma, inferior vena cava, complications, surgical choice

## Abstract

**Objective:**

To explore the role of tumor volume (TV) on surgical approach choice, surgical complexity, and postoperative complications in patients with renal cell carcinoma (RCC) and inferior vena cava tumor thrombus.

**Method:**

From January 2014 to January 2020, we retrospectively analyzed the clinical data of 132 patients who underwent radical nephrectomy with inferior vena cava thrombectomy (RN-IVCT). Primary renal tumor volume (PRTV), renal vein tumor thrombus volume (RVTTV), inferior vena cava tumor thrombus volume (IVCTTV), and total tumor thrombus volume (TTTV) were measured with the help of an internationally recognized 3D volume measurement software. The patients were divided into three groups according to the tumor volume within the inferior vena cava (IVC). Group 1 included 48 patients with IVCTTV between 0 and 15 cm3 (36.6%), group 2 included 38 patients with IVCTTV between 16 and 30 cm3 (28%), and group 3 included 46 patients with IVCTTV above 30 cm3 (35%). The three IVCTTV groups, as well as four different volume groups, were compared in terms of surgical approach choice, surgical complexity, and postoperative complications. One-way ANOVA and a non-parametric test were used to compare the clinicopathological characteristics and distribution differences between the three groups.

**Result:**

This study found significant differences among the three groups in the proportion of open surgery (P < 0.001), operation time (P < 0.044), intraoperative bleeding (P < 0.001), and postoperative complications (P < 0.001). When the four different volumes were compared, we found that for higher volumes IVCTTV and TTTV, open surgery is used more often compared with laparoscopic surgery (P < 0.001). In addition, with the increase in renal vein tumor thrombus volume, inferior vena cava tumor thrombus volume, and total tumor thrombus volume, the operation time also increased. Finally, with the increase in tumor thrombus volume and total tumor thrombus volume, the amount of intraoperative bleeding increased.

**Conclusion:**

With the increase in tumor volume, the proportion of open surgery and the incidence of postoperative complications increased. In addition, larger tumor volume prolongs operation time, increases intraoperative blood loss, and makes the surgery more complicated.

## Introduction

Renal cell carcinoma (RCC) is one of the most common malignant tumors of the urinary system, accounting for 3%–4% of adult malignant tumors (1, 2). Around 4%–10% of the patients with renal cell carcinoma invade the renal vein (RV) and inferior vena cava (IVC) to form tumor thrombus (3, 4). Although Berg performed the first radical nephrectomy combined with inferior vena cava thrombectomy (RN-IVCT) for the first time in 1913 (5), this surgery is still one of the most complex and challenging operations in urology. Therefore, many studies were conducted on the effects of tumor size, tumor thrombus grade level, adrenal involvement, lymph node, and distant metastasis on the prognosis and surgical planning for these patients. However, the significance of tumor volume (TV) has not been evaluated for RCC patients with inferior vena cava tumor thrombus (6).

Many classifications have been introduced since Berg performed the first ever RN-IVCT in 1913 (7–10). Among them, many surgeons use the traditional Mayo grade classification for the surgical planning. Although this is a very useful classification, there are some limitations, such as not considering the width and IVC vascular wall invasion, which may lead to a poor evaluation of the surgical planning and prognosis of these patients. Therefore, we aim to evaluate the surgical approach choice, surgical complexity, and postoperative complications according to the tumor thrombus volume, in the hope to help surgeons choose the better surgical approach, evaluate the complexity of the operation reasonably, effectively avoid postoperative complications, and pay more attention to the patients with higher postoperative risk.

## Materials and Method

### Patients

**Figure 1:**
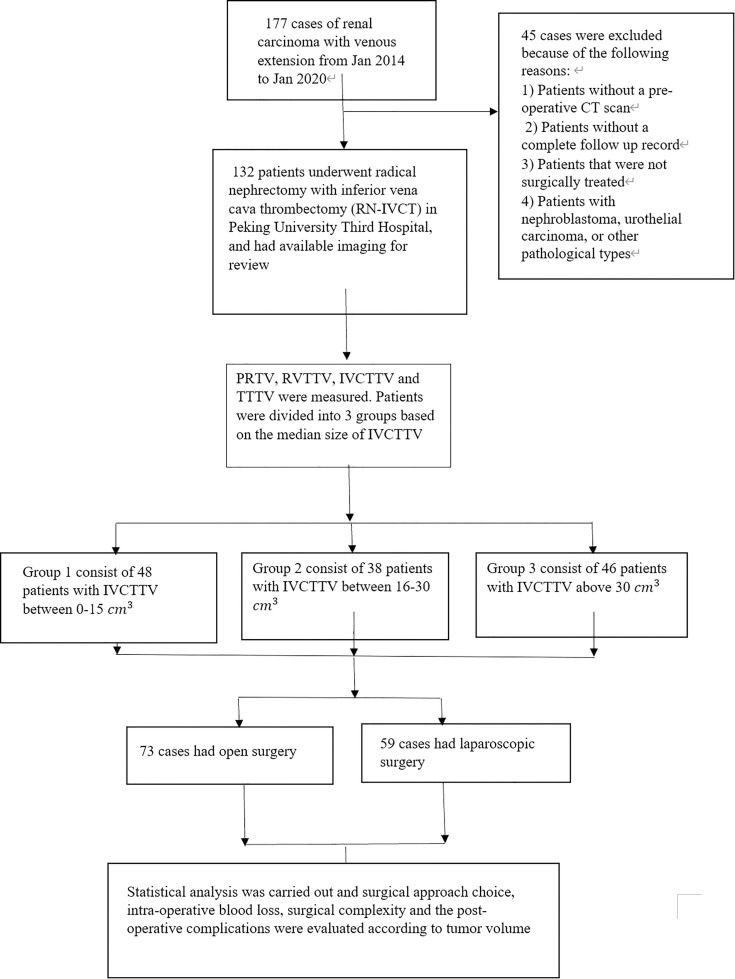
Flowchart of this study.

The data of 177 patients that underwent RN-IVCT between January 2014 to January 2020 at our center were collected. After excluding unqualified patients, a total of 132 patients were included in this study. The exclusion criteria of this study were 1) patients that were not surgically treated; 2) nephroblastoma, urothelial carcinoma, or other pathological types of tumors; 3) patients without a preoperative CT; and 4) patients with incomplete follow-up records (**Figure 1**).

### Tumor Volume Measurements

In this study, the 3D tumor volume of 132 patients was measured by using the latest version of 3D Slicer. 3D Slicer is a free and open-source 3D visual medical image processing and information analysis software. The application was first created by David Gering in 1999 and later developed by the National Institutes of Health and the global developer community (11, 12). The software has been used and cited in more than 443 publications and more than 36 lung-specific articles (12).

**Figure 2:**
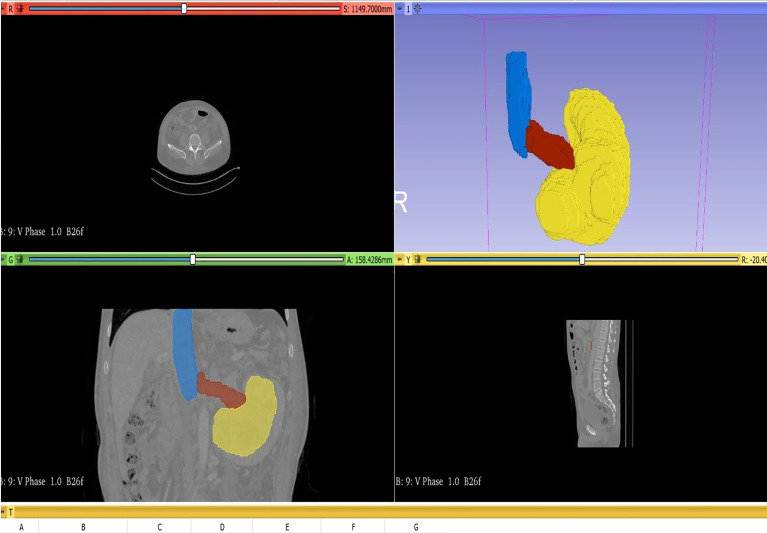
Method of measuring tumor volume using 3D Slicer. Yellow: PRTV; red: RVTTV; blue: IVCTTV.

In this study, to measure the tumor volume, two urologists and one radiologist evaluated the tumor size based on coronal and sagittal images of abdominal CT. To measure the volume, we first have to import the original DICOM format data of the patient’s abdominal CT into the 3D Slicer software. Next, use the segment editor module to reconstruct the CT data, open show 3D, click surface smoothing in its submenu, and adjust the smoothing factor to the maximum value of 1.0 to make the reconstructed image surface smoother. Then click the add button to select the color (yellow: primary renal tumor, red: renal vein tumor thrombus, blue: inferior vena cava tumor thrombus, as shown in **Figure 2**), click paint and carefully color each slice of the tumor, and observe the reconstruction effect while painting. Next, using the measurement tool, plot each axial section of the primary renal tumor volume, renal vein tumor thrombus volume, and inferior vena cava tumor thrombus, which provides an area in cubic centimeters (cm3) for each cut. Carefully measure the segmental volumes between each slice and add to calculate the final tumor volume.

### Volume Grouping

**Figure 3:**
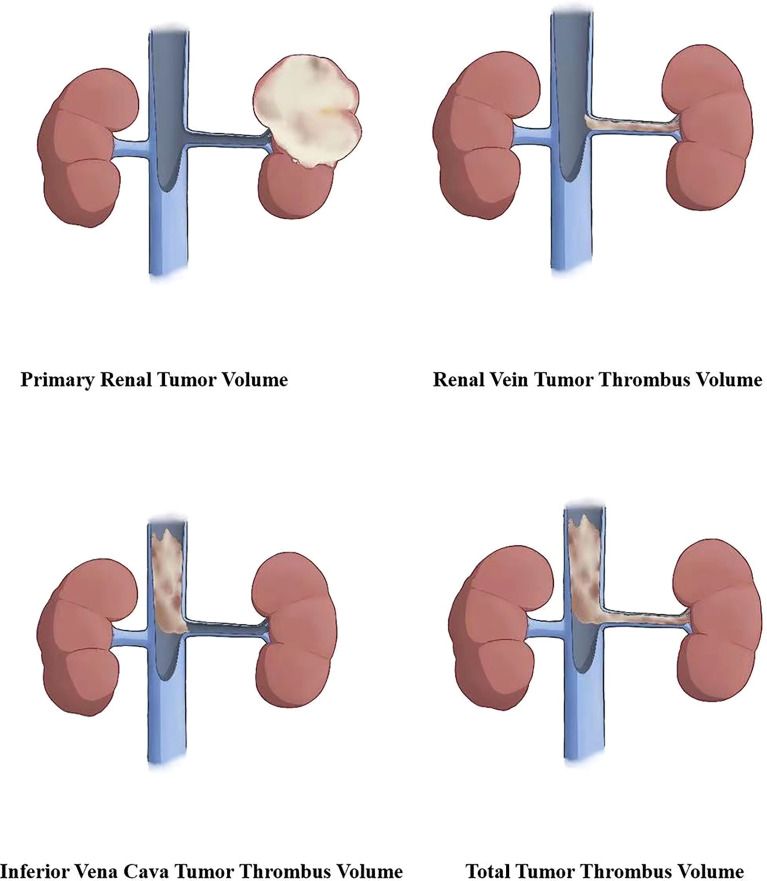
According to the anatomical location of the renal carcinoma and inferior vena cava tumor thrombus, we measured the primary renal tumor volume (PRTV), renal vein tumor thrombus volume (RVTTV), and inferior vena cava tumor thrombus volume (IVCTTV). We obtained the total tumor volume (TTTV) by adding RVTTV and IVCTTV.

According to the anatomical location of the renal cell carcinoma and inferior vena cava tumor thrombus, primary renal tumor volume (PRTV), renal vein tumor thrombus volume (RVTTV), and inferior vena cava tumor thrombus volume (IVCTTV) were measured. Total tumor thrombus volume (TTTV) was obtained by adding renal vein tumor thrombus volume and inferior vena cava tumor thrombus volume (TTTV = RVTTV + IVCTTV), as shown in **Figure 3**.

**Figure 4:**
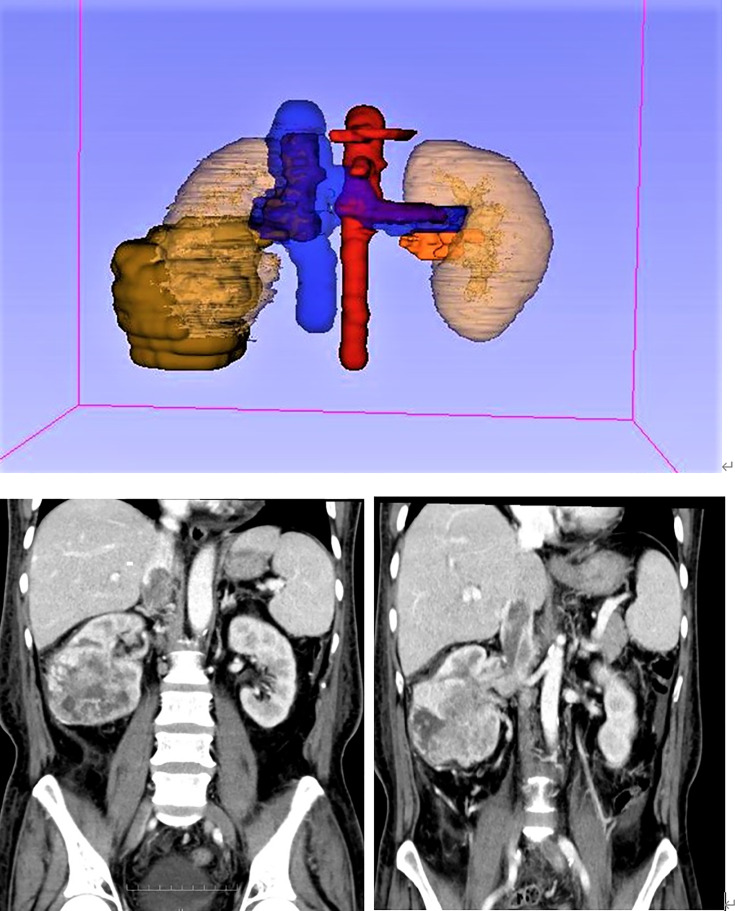
A 3D figure of a 53-year-old Chinese male patient made with 3D Slicer.

The patients were also divided into three groups according to the median size of IVCTTV. Group 1 included 48 patients with IVCTTV between 0 and 15 cm3 (36.6%), group 2 included 38 patients with IVCTTV between 16 and 30 cm3 (28%), and group 3 included 46 patients with IVCTTV above 30 cm3 (35%) (**Figure 4**).

### Follow-up

All the patients were evaluated for postoperative recurrence and general condition with abdominal CT or magnetic resonance imaging (MRI) every 3 months after the surgery in the first year, and then every 6 months afterward. Chest CT and brain MRI were used to detect metastasis. Postoperative complications were graded according to the Clavien–Dindo classification. Grade I–II events are classified as minor complications, and grade III–V events are classified as major complications. All complications were recorded within 30 days. Follow-up information was obtained *via* phone interviews and outpatient records.

### Statistical Analysis

Operation time, operation complexity, surgical approach choice, intraoperative bleeding, IVC wall invasion, and Mayo grade level were compared between the three groups. In addition, the differences in age, sex, side, body mass index, pathological type, renal sinus fat infiltration, and metastasis were compared among three groups. All statistical analyses were performed using the SPSS software version 23. Continuous variables were tested for normality. One-way ANOVA was used to compare the clinicopathological features of the three groups. For the data with abnormal distribution, the median (minimum and maximum) and non-parametric test were used to compare the distribution differences among the groups. A value of P < 0.05 was considered statistically significant.

## Results

The patient characteristics are shown in **Table 1**. The patients were divided into three groups according to the median size of IVCTTV. Group 1 included 48 patients with IVCTTV between 0 and 15 cm3 (36.6%), group 2 included 38 patients with IVCTTV between 16 and 30 cm3 (28%), and group 3 included 46 patients with IVCTTV above 30 cm3 (35%). All the patients had tumor thrombus extending to the RV and IVC and underwent surgical treatment (36 left-sided and 96 right-sided). No significant difference was noted for demographic characteristics such as age, gender, side, size, BMI index, and tumor type among the three groups.

**Table 1:** Patient characteristics.

	Group 1	Group 2	Group 3	P
Gender, n (%) Male Female	31 (64.6) 17 (35.4)	30 (78.9) 8 (21.1)	39 (84.7) 7 (15.3)	0.063
Age, y, mean ± SD	59.13 ± 9.795	62.05 ± 10.77	58.30 ± 8.167	0.182
BMI, kg/*m* ^2^ median (IQR)	22.40 (16.36, 32.89)	23.37 (17.93, 33.03)	24.8 (15.23, 33.03)	0.054
Side, n (%) Left Right	16 (33.3) 32 (66.7)	9 (23.7) 29 (76.3)	11 (23.9) 35 (76.1)	0.497
Lymph node metastasis, n (%) No Yes	17 (35.4) 31 (64.6)	15 (39.5) 23 (60.5)	14 (30.4) 32 (69.6)	0.684
Ipsilateral adrenalectomy, n (%) No Yes	45 (93.8) 3 (6.3)	32 (84.2) 6 (15.8)	41 (89.1) 5 (10.9)	0.360
Distant metastasis, n (%) No Yes	33 (68.8) 15 (31.3)	26 (68.4) 12 (31.6)	32 (69.6) 14 (30.4)	0.993
Presence of bland thrombus, n (%) No Yes	38 (79.2) 10 (20.8)	31 (91.6) 7 (18.4)	28 (60.9) 18 (39.1)	0.054
Renal sinus fat infiltration, n (%) No Yes	41 (89.1) 5 (10.9)	34 (89.5) 4 (10.5)	40 (87.0) 6 (13.0)	0.923
Perirenal fat infiltration, n (%) No Yes	37 (77.1) 11 (22.9)	25 (65.8) 13 (34.2)	29 (63.0) 17 (37.0)	0.300
Pathology type, n (%) Clear cell RCC Non-clear cell RCC	40 (83.3) 8 (16.7)	28 (73.7) 10 (26.3)	38 (82.6) 8 (17.4)	0.476
Surgical approach, n (%) Laparoscopic surgery Open surgery	32 (66.7) 16 (33.3)	19 (50.0) 19 (50.0)	8 (17.4) 38 (82.6)	**0.001**
Mayo classification, n (%) I II III IV	15 (31.3) 28 (53.8) 3 (6.3) 2 (4.2)	5 (13.2) 25 (65.8) 4 (10.5) 7 (10.5)	4 (8.7) 21 (45.7) 12 (26.1) 9 (19.6)	**0.002**
Operative time, min, median (IQR)	334 (165, 589)	341.5 (161, 796)	374 (219, 873)	**0.044**
Surgical blood loss, mL, median (IQR)	650 (20, 4,500)	600 (20, 4,700)	2,350 (0, 8,800)	**0.001**
Intra-operative blood transfusion, mL, median (IQR)	0 (0, 800)	400 (0, 1,600)	1,500 (700, 2,500)	**0.008**
Postoperative blood transfusion, mL, mean ± SD	17 ± 81	21 ± 91	130 ± 280	**0.001**
IVC wall invasion, n (%) No Yes	31 (70.5) 13 (29.5)	16 (45.7) 19 (54.3)	15 (36.6) 26 (63.4)	**0.005**
Liver mobilization, n (%) No Yes	42 (87.5) 6 (12.5)	30 (78.9) 8 (21.1)	28 (60.9) 18 (39.1)	**0.009**
Extracorporeal circulation, n (%) No Yes	46 (95.8) 2 (4.2)	38 (100) 0 (0.00)	40 (87.0) 6 (13.0)	**0.018**
Postoperative hospital stays, median (IQR)	8 (4.34)	9 (4.61)	10 (4.70)	0.089
IVC tumor diameter, cm, mean ± SD	2.44 ± 0.88	2.96 ± 0.80	3.46 ± 0.88	**<0.001**

Significant difference was noted in terms of surgical approach choice, Mayo classification, operative time, surgical blood loss, intraoperative and postoperative blood transfusion, IVC wall invasion, liver mobilization, extracorporeal circulation, and IVC tumor diameter.

Bold values means theres is an statistical difference.

Overall, 59 patients (44.7%) underwent complete laparoscopic surgery and 73 patients (55%) underwent open surgery. The proportion of open surgery increased in patients with larger IVCTTV (P < 0.001). As shown in **Table 1**, 32 patients (66.7%) underwent complete laparoscopic surgery and 16 patients (33.3%) underwent open surgery in group 1. In group 2, 19 patients (50%) underwent laparoscopic surgery and 19 patients (50%) underwent open surgery. Finally, only 8 patients (17.6%) underwent laparoscopic surgery and 38 patients (82.6%) underwent open surgery in group 3. In addition, the number of patients that needed liver mobilization (6 vs. 8 vs. 18, P <0.009) and extracorporeal circulation (2 vs. 0 vs. 6, P < 0.018) increased with an increase in IVCTTV (**Table 1**). In terms of surgical approach choice among the four different anatomical tumor volumes, we found that for higher volumes IVCTTV and TTTV, open surgery is used more often compared with laparoscopic surgery, while no significant difference was noted in PRTV and RVTTV, as shown in **Table 2**.

**Table 2:** Comparison between median PRTV, RVTTV, IVCTTV, and TTTV in terms of surgical approach choice.

	Laparoscopic approach	Open approach	P
PRTV, median (IQR)	216.9 (22.0, 639.1)	236.71 (7.66,1532.1)	0.332
RVTTV, median (IQR)	9.1 (1.9, 35.6)	9.6 (1.42, 39)	0.741
IVCTTV, median (IQR)	14.6 (2.9, 46.2)	32.26 (3.6, 103.41)	**0.001**
TTTV, median (IQR)	27.4 (7.3, 65.6)	43.5 (7.5, 114.027)	**0.001**

For higher volumes IVCTTV and TTTV, open surgery is used more often compared with laparoscopic surgery. PRTV and RVTTV size does not influence the operative method.

Bold values means theres is an statistical difference.

In terms of operative time, it was prolonged along with the IVCTTV (P < 0.044). As shown in **Table 1**, the median operation time was 334, 341, and 374 min for groups 1, 2, and 3, respectively. When we compared the four different anatomical tumor volumes using linear regression, we found that operative time was prolonged with the increase in RVTTV, IVCTTV, and TTTV (**Table 3a**).

**Table 3A:** Linear regression analyses—comparison between PRVT, PRVTTV, IVCTTV, and TTTV in terms of operation time and surgical complexity.

	P	95% CI	β
PRVT	0.799	-0.009 (-0.083, 0.064)	-0.023
RVTTV	**0.014**	3.362 (0.693–6.031)	0.216
IVCTTV	**0.033**	1.167 (0.094–2.239)	0.188
TTTV	**0.005**	1.373 (0.422–2.323)	0.246

CI, confidence interval; β, standardized β coefficient.

Data are expressed as standardized beta coefficients that were tested by linear regression analysis. With the increase in size of renal vein tumor thrombus volume, inferior vena cava tumor thrombus volume, and total tumor thrombus volume, the operation time and surgical complexity increased.

Bold values means theres is an statistical difference.

Intraoperative blood loss increased along with the IVCTTV (P < 0.001). The median intraoperative blood loss was 650, 600, and 2350 ml in groups 1, 2, and 3, respectively (P < 0.001). In addition, median intraoperative (P < 0.008) and mean postoperative blood transfusion (P < 0.001) increased along with the size of IVCTTV. When comparing the four different anatomical tumor volumes using linear regression, we found that intraoperative blood increased with the increase in IVCTTV and TTTV (**Table 3b**).

**Table 3B:** Linear regression analyses—comparison between PRTV, RVTTV, IVCTTV, and TTTV in terms of surgical bleeding.

	P	95% CI	Coefficient
PRTV	0.142	0.757 (-0.258, 1.772)	0.128
RVTTV	0.434	14.473 (-22.027–50.974)	0.069
IVCTTV	**0.001**	32.185 (18.847–45.523)	0.386
TTTV	**0.001**	27.317 (15.346–39.293)	0.368

CI, confidence interval; β, standardized β coefficient.

Data are expressed as standardized beta coefficients that were tested by linear regression analysis. With the increase in the size of tumor thrombus volume and total tumor thrombus volume, the amount of intraoperative bleeding increased.

Bold values means theres is an statistical difference.

Postoperative complications were recorded in 63 patients. The incidence of postoperative complications was significantly higher in group 3 compared to the other two groups (P < 0.001, 27% vs. 40% vs. 76%). Complications were also graded according to the Clavien–Dindo classification. Grade I–II events are classified as minor complications, and grade III–V events are classified as major complications. All complications were recorded within 30 days. A significant difference was noted in minor complications (P < 0.001), while no significant difference was noted in major complications (**Table 4**).

**Table 4:** Complications.

Complications	Grade	Incidence rate			*P*
n = 63		IVCTTV group 1	IVCTTV group 2	IVCTTV group 3	* *
Minor	I	1	0	4	0.001
	II	9	10	25	
					
Major	III	0	1	0	0.34
	IVa	2	2	6	
	IVb	0	1	0	
	V	1	1	0	

Complications were graded according to the Clavien–Dindo classification. Grade I–II events are classified as minor complications, grade III–V events are classified as major complications. All complications were recorded within 30 days.

**Figure 5:**
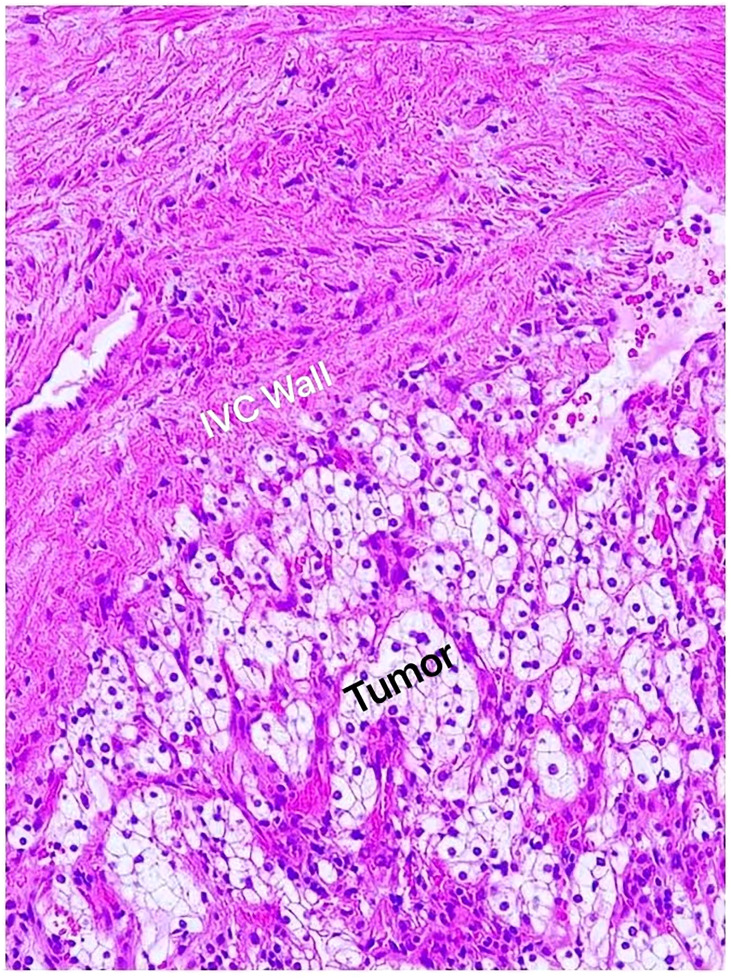
Histological examination confirmed renal cell carcinoma; clear cell type (Fuhrman grade II) invading the IVC vascular wall.

Overall, 58 patients were found to have tumors invading the IVC vascular wall. IVC wall invasion was confirmed with histological examination (**Figure 5**). Incidence of the IVC wall invasion was found to be higher in group 3 (P < 0.005, 13 vs. 19 vs. 26). No significant difference was noted when we compared the incidence of IVC wall invasion and the Mayo grade (**Table 5**).

Table 5AComparison between Mayo grade level and incidence of IVC wall invasion. No significant difference noted.

**Table:** 

Mayo grade	With IVC wall invasion	Without IVC wall invasion	*P*
1	17	7	0.102
2	32	32	
3	6	12	
4	7	6

**Table 5B:** Comparison between IVCTTV groups and incidence of IVC wall invasion.

IVC wall invasion	IVCTTV group 1	IVCTTV group 2	IVCTTV group 3	P
No Yes	31 (70.5) 13 (29.5)	16 (45.7) 19 (54.3)	15 (36.6) 26 (63.4)	**0.001**

A significant difference was noted among the three groups. With the increase of IVCTTV, the incidence of IVC wall invasion increased.

Bold values means theres is an statistical difference.

There were 24 patients with Mayo grade I, 74 patients with Mayo grade II, 19 patients with Mayo grade III, and 18 patients with Mayo IV. The number of patients with Mayo grade I was 15 (31.3%), 5 (13.2%), and 4 (8.7%) for groups 1, 2, and 3, respectively. The number of patients with Mayo grade II was 28 (53.8%), 25 (65.8%), and 21 (45.7%) for groups 1, 2, and 3, respectively. The number of patients with Mayo grade III was 3 (6.3%), 4 (10.5%), and 12 (26.1%) for groups 1, 2, and 3, respectively. Lastly, the number of patients with Mayo IV was 2 (4.2%), 7(10.5%), and 9 (19.6%) for groups 1, 2, and 3, respectively (P < 0.002).

## Discussions

This study aims at exploring the role of TV within the inferior vena cava, and four different anatomical tumor volumes (PRTV, RVTTV, IVCTTV, and TTTV) on surgical approach choice, surgical complexity, and postoperative complications for patients undergoing RN-IVCT. We used the 3D Slicer volume-measuring application to measure the 3D tumor volumes because previous studies suggested that 3D volume is more accurate compared to volumes calculated with π/6 (length × width × height), as most solid tumors are not regular spheres or ellipsoids (13). 3D Slicer is an internationally recognized 3D medical image processing and information analysis software. This application was first created by David Gering in 1999 and was developed by the National Institutes of Health and the global developer community (11). This software has been used and cited in more than 443 publications and more than 36 pulmonary-specific articles (12).

Radical nephrectomy combined with resection of inferior vena cava tumor thrombus is the best treatment choice for renal carcinoma with inferior vena cava tumor thrombus (2), but very little is known about the best surgical approach, and the studies on this particular aspect are limited. Although many classifications have been introduced since Berg performed the first ever RN-IVCT in 1913 (7–10), all of them are used for open surgery (14). At present, many surgeons use the Mayo classification for the surgical planning and prognosis of these patients. Although Mayo classification is particularly useful for measuring the height of the IVC tumor thrombus, the significance of tumor width and IVC vascular wall invasion is barely taken into consideration, which can be insufficient to evaluate the surgical approach choice for patients with a wide tumor, but a low-grade Mayo level. To reduce the incidence of intraoperative and postoperative complications, such as more intraoperative blood loss, longer operative time, and postoperative wound-related infection, it is of cardinal importance to know and select an ideal surgical approach for every patient. In this study, we found that IVCTTV has an important role in the choice of surgical approach. We found a reduction in the number of patients who underwent laparoscopic surgery when IVCTTV was greater than 30 cm3. Even in those few cases where laparoscopic surgery was attempted, the chances of converting to open surgery were high.

In addition, this study found that intraoperative blood loss increased along with the size of IVCTTV. The median blood loss in group 1 (IVCTTV <15 cm3) was 650 ml. This number rose to 2,350 ml for the patients with IVCTTV >30 cm3. A study by Zarger et al. reached a similar conclusion (6). In addition, median intraoperative blood transfusion, as well as mean postoperative blood transfusion, increased with the size of IVCTTV. In terms of postoperative complication, we found that a higher incidence of postoperative complications was observed in group 3, in comparison to group 1 and group 2 (13 vs. 15 vs. 35, P < 0.001). However, when we ranked complications according to Clavien–Dindo, a significant difference was noted in minor complications, while no significant difference was noted in major complications among the groups. Evaluating surgical bleeding and complications according to the tumor volume can help surgeons make full preparation before the operation to reduce the incidence of possible complications. It is also very important that for patients with IVCTTV above 30 cm3, sufficient blood and an intensive care unit (ICU) ward should be prepared, and patients should be informed about complications associated with IVCTTV size before the surgery.

Other important factors associated with TV were the depth of IVC vascular wall invasion, tumor diameter, and Mayo grade. According to previous studies, IVC vascular wall invasion and width of IVCT can significantly increase the difficulty of surgery and is associated with a poor prognosis in RCC patients with venous extension (15, 16). Therefore, it is very beneficial to consider both width and height of tumor thrombus during surgical planning. However, the Mayo grading system, which is widely used for surgical planning of these patients, does not take tumor width and IVC vascular invasion into consideration. This could be insufficient for the evaluation of the surgical approach choice, estimation of blood loss, and incidence of postoperative complications for patients with a wide tumor, but a low Mayo grade. In this study, to better explore the role of TV on the incidence of IVC wall invasion, we separately evaluated the correlation between Mayo grade and IVC wall invasion, as well as that between TV and IVC wall invasion. We found a significant difference among the three IVCTTV groups (P < 0.001), while no significant difference was noted when we compared the Mayo grade and the incidence of IVC vascular wall invasion (P < 0.102). A previous study conducted by Alayed et al. found that TV can be an excellent indicator of IVC wall invasion (17). In addition, we found that there are several patients with a Mayo grade I tumor, who have similar operative times, similar intraoperative blood loss, and similar incidence of postoperative complications as those with Mayo grade III or IV. We believe that the reason for this is that a wide tumor with a large volume is associated with increased operation time, increased surgical complexity, more intraoperative blood loss, and a higher incidence of postoperative complications. Therefore, for patients with a low Mayo grade tumor but large IVCTTV, similar measures, such as preparation of sufficient blood and an ICU ward, should also be taken.

In terms of prognosis, some studies found that the level of tumor thrombus is an independent prognostic factor for survival (18, 19); however, other studies did not find that the level of tumor thrombus is an independent prognostic factor for survival (20–25). In this study, the association between the tumor volume and overall survival was not observed. Zarger et al.’s study reported that tumor volume >15 cm3 is associated with a poor OS in RCC patients with IVC tumor thrombus (6). However, limited follow-up data and relatively small cohort size (66 patients) were among their limitations. After related literature revision, we found that a previous study about esophageal cancer conducted by Créhange et al. reported that 100 cm3 was the optimal cutoff value to distinguish OS (26). In addition, Chen et al.’s research on tumor volume association with esophageal cancer results showed that the cutoff value of tumor volume was 20 cm3 (27). We believe that further studies with larger numbers are required to clarify the association between TV and OS for RCC patients with IVC tumor thrombus.

This study has some limitations. First, all patients underwent surgery in the same hospital, so similar techniques were used. In addition, although two experienced urologists and one radiologist measured the tumor volume, the measurement results may not be as accurate as the automatic measurement of artificial software. However, the advantages of this study are the number of patients than in previous studies and this study being the very first to perform a comparative analysis on the significance of tumor volume on operation method, intraoperative bleeding, postoperative complications, and survival rate in RCC patients with venous extension. These results can help surgeons choose the correct operation method according to the size of tumor volume, predict intraoperative blood loss and postoperative complications, and make full preparation before operation. At the same time, this study also reflects the significance of tumor volume and provides a certain basic significance for the development of automatic tumor volume measurement software within MRI machine in the future.

## Conclusion

With the increase in tumor volume, the proportion of open surgery and the incidence of postoperative complications increased. In addition, larger tumor volume prolongs operation time, increases intraoperative blood loss, and makes the surgery more complicated.

## Data Availability Statement

The raw data supporting the conclusions of this article will be made available by the authors, without undue reservation.

## Ethics Statement

The studies involving human participants were reviewed and approved by the Ethics Committee of Peking University Third Hospital. The patients/participants provided their written informed consent to participate in this study. Written informed consent was obtained from the individual(s) for the publication of any potentially identifiable images or data included in this article.

## Author Contributions

QB, ZL and YCH contributed as the first authors of this study. QB and R.R.R.S measured the tumor volume using 3D Slicer and made figures. YXL analyzed the statistical data. XZ provided the clinical imaging data. PH made the literature review. ZLW performed histological examinations. LLM made the final revision. All authors contributed to the design of this project, data collections, and literature review. All authors have read and approved the final submitted manuscript. 

## Conflict of Interest

The authors declare that the research was conducted in the absence of any commercial or financial relationships that could be construed as a potential conflict of interest.

The reviewer YF declared a shared parent affiliation with the authors to the handling editor at the time of review.

## Publisher’s Note

All claims expressed in this article are solely those of the authors and do not necessarily represent those of their affiliated organizations, or those of the publisher, the editors and the reviewers. Any product that may be evaluated in this article, or claim that may be made by its manufacturer, is not guaranteed or endorsed by the publisher.
